# A Low-Cost Radar-Based IoT Sensor for Noncontact Measurements of Water Surface Velocity and Depth

**DOI:** 10.3390/s23146314

**Published:** 2023-07-11

**Authors:** Stephen Catsamas, Baiqian Shi, Miao Wang, Jieren Xiao, Peter Kolotelo, David McCarthy

**Affiliations:** 1 BoSL Water Monitoring and Control, Department of Civil Engineering, Monash University, Melbourne, VIC 3800, Australia; stephen.catsamas@monash.edu (S.C.); baiqian.shi@monash.edu (B.S.); miao.wang@monash.edu (M.W.); peter.kolotelo@monash.edu (P.K.); 2 School of Civil and Environmental Engineering, Faculty of Engineering, Queensland University of Technology, Brisbane, QLD 4001, Australia

**Keywords:** Doppler radar, field verification, IoT, low cost, low power, open hardware, noncontact, radar level measurement, real-time environmental monitoring, sensor design, stormwater monitoring, urban water, water depth, water velocity

## Abstract

We designed an out-of-water radar water velocity and depth sensor, which is unique due to its low cost and low power consumption. The sensor is a first at a cost of less than USD 50, which is well suited to previously cost-prohibited high-resolution monitoring schemes. This use case is further supported by its out-of-water operation, which provides low-effort installations and longer maintenance-free intervals when compared with in-water sensors. The inclusion of both velocity and depth measurement capabilities allows the sensor to also be used as an all-in-one solution for flowrate measurement. We discuss the design of the sensor, which has been made freely available under open-hardware and open-source licenses. The design uses commonly available electronic components, and a 3D-printed casing makes the design easy to replicate and modify. Not before seen on a hydrology sensor, we include a 3D-printed radar lens in the casing, which boosts radar sensitivity by 21 dB. The velocity and depth-sensing performance were characterised in laboratory and in-field tests. The depth is accurate to within ±6% and ±7 mm and the uncertainty in the velocity measurements ranges from less than 30% to 36% in both laboratory and field conditions. Our sensor is demonstrated to be a feasible low-cost design which nears the uncertainty of current, yet more expensive, velocity sensors, especially when field performance is considered.

## 1. Introduction

When monitoring, modelling, and engineering urban water systems, one critical hydrological variable is the volumetric flowrate or discharge. Knowledge of discharge is important in optimising future urban water systems and minimising their environmental impact [1,2], detecting and locating leaks and burst pipes [3], and estimating pollutants from combined sewer overflow events [4,5]. Flow can be measured in a number of ways, ranging from manual dilution methods (i.e., adding salt tracers and EC sensors [6]), using stage discharge relationships that may or may not include the use of hydraulic control structures (i.e., weirs [7,8,9]), to the velocity–area method. Due to its accuracy and relative ease of installation, the velocity–area method is by far the most common method for small streams and stormwater drains. The cross-sectional area can be estimated from a water depth measurement using known bathymetry [10]. The mean flow velocity can either be obtained from in-water sensors or estimated via the surface velocity from an out-of-water sensor [9,11,12].

To facilitate a deeper understanding and to obtain a more accurate understanding of waterways, higher resolution monitoring has been desired so that real-time monitoring and control of our urban waterways can be achieved [3,13,14,15]. For flowrate monitoring via the velocity–area method, this means having suitable velocity and depth sensors. Unfortunately, commercially available sensors for monitoring velocity, such as QCam [16] or the HACH AV sensor [17], are expensive, with per-sensor costs of several thousand USD, onerous to install, and require ongoing maintenance [18,19]. This limits their potential for deployment in the large quantities needed for higher resolution monitoring and prompts the call for low-cost, low-maintenance sensors that are easy to install [20].

Fortunately, low-cost velocity sensing has seen some advances in recent years. Particularly, in-water sensors have been developed, such as the hydromast proposed in [21], which utilises the pressure exerted by the water flow to measure the water velocity at the bed of the channel. A low-cost acoustic Doppler sensor is proposed in [22] and uses continuous-wave ultrasonic Doppler to obtain a measurement for the water velocity.

While low-cost sensors have shown promise in accurately and inexpensively measuring water velocity, these in-water sensors need to be located within the water stream [21,22]. In-water sensors are susceptible to debris and may require hazardous confined-space entry to be mounted on the channel bed [23]. This can lead to additional costs, initially during installation and in maintenance over the lifetime of the sensor. For example, [24] outlines the severe challenges of operating an in-water sensor, including maintenance up to every two weeks, which is not logistically or economically possible when considering a dense network of sensors in a water catchment. Out-of-water and noncontact sensors promise to offer the same measurements, but without contact with the body of water being measured, hence alleviating these issues.

There are plenty of examples that describe how to measure water depth using noncontact, out-of-water approaches [25,26]. An advanced approach was described by [27], who developed a novel out-of-water depth sensor by measuring the phase of a reflected radar signal. This approach achieved millimetre accuracy; however, it is unclear if it would be suitable for use in remote-area applications given power and size constraints. Unfortunately, there are a limited number of examples in the literature of noncontact velocity sensing, and those that exist in the commercial realm have high purchase costs.

There are two technologies reported in the research literature for out-of-water velocity sensing: particle image velocimetry and Doppler radar velocimetry [28,29]. Particle image velocimetry techniques, while delivering accurate results, are not well adapted to low-power or low-cost sensing due to their use of computationally heavy algorithms [30,31,32] which require high battery power and processors sufficiently adapted to the required calculations. Additionally, image-based techniques are susceptible to adverse weather, such as fog, or lighting conditions, such as nighttime [33]. Doppler radar has recently been shown to be viable as a low-cost technology in [34], in which a prototype custom radar sensor and data processing algorithm was developed. These authors failed to develop an autonomous ‘field-ready’ sensor and simply demonstrated that the necessary technologies are feasible. The authors of [35] utilised a custom-designed low-noise amplifier integrated circuit, which is cost-infeasible for all but commercial scales. They do, however, incorporate an inclinometer for automatic measurement of the sensor angle. Neither of these works investigated combining depth measurement into the sensor.

To the authors’ knowledge, there have only been two recent works discussing a combined water level and velocity sensor. Both [36,37] use in-water sensors that use optical properties to determine the water level and velocity. These papers focus on the proof of concept of the measurement principle and do not consider their incorporation into a ‘field-ready’ or low-cost design.

It is apparent that current approaches to low-cost velocity sensing each have their own limitations. Our review of the literature on the current state of low-cost sensors concluded that there is not a current viable solution for low-cost velocity and depth monitoring. This paper proposes a newly designed sensor to address this significant gap in the space of low-cost water monitoring.

The sensor presented in our paper is a low-cost, low-power, out-of-water radar sensor that can measure both water velocity and water depth. It addresses many of the challenges found when using existing sensors. By being both low-power and out-of-water, the sensor is low-maintenance as its batteries need to be changed very infrequently, and being out of the water, it is less likely to be blocked by debris. Furthermore, being out-of-water also simplifies the installation of the sensor, as mounting the sensor above the waterway can often be accomplished without entering the waterway or requiring confined-space entry.

While previous works have explored the potential of low-cost radar measurement, they left significant work in terms of bringing this technology into a state ready for use is large-scale environmental monitoring schemes. In contrast, the sensor developed in this study is ready for field operation in such monitoring schemes. Our sensor advances the out-of-water noncontact sensing space by integrating depth and velocity sensing into a single package, utilises 3D printing for cost reduction and reproduction ease, and includes a 3D-printed radar lens to vastly increase the sensitivity of the device.

In this paper, (1) the design and operating principle of the sensor are discussed, (2) the sensor is validated and characterised in laboratory and in-field tests, and (3) the results of the laboratory and in-field tests are used to characterise the sensor and analyse its performance.

## 2. Materials and Methods

### 2.1. Sensor Design

The sensor uses radar to measure both the water velocity and the water depth. A commercial off-the-shelf radar chip, the Acconeer XM132 [38], handles the generation and detection of the radar signals. The XM132 uses a pulsed coherent 60 GHz radar. In general, radar operates by emitting a radio wave which then reflects off an object and is detected by the radar antenna. Pulsed coherent radar sends out short pulses of radar, the time of flight of which can be used to determine the distance to the object. As the pulses are coherent, their phase is well known. This can be used to determine the phase upon reflection, which can encode velocity information via the Doppler effect. For the context of water velocity measurement, the radar is typically reflected back to the sensor due to the effect of Bragg scattering. This occurs off short surface waves with wavelength on the order of the radar wavelength, which may be caused by wind, rain, turbulence, and longer surface waves [39,40]. These surface waves travel at a speed of a few tens of centimetres per second along the water surface, hence in general the reflected signal contains multiple frequency components from the advancing and receding surface waves. Due to current variations, these two frequency components may have differing amplitudes and may not be distinguishable. Ideally, the midpoint of the two frequency components should be measured to obtain the surface velocity [39,40]. The Doppler effect then defines the relationship between the frequency shift of the radar upon reflection and the water velocity via

(1) $$v=\frac{c\Delta f}{2f\cos\theta }$$

where *c* is the speed of light in air, Δ*f* is the frequency shift between incoming and outgoing radar signals, *f* is the radar frequency (fixed for the used radar module), and *θ* is the angle between the radar and object motion.

The line-of-sight distance to the water surface can be determined by considering the time taken for the radar signal to return to the sensor. Therefore, the distance *d* to the water surface can be determined using the following:

(2) $$d=\frac{ct}{2}$$

where *t* is the time of flight of the pulse.

For a reflection off a water surface, a radar measurement can determine both the Doppler shift and the time of flight. Here, as also seen in [35], it is approximated that the water surface is perpendicular to gravity, hence an accelerometer mounted to the radar sensor can be used to determine *θ*. Once this is known, Equations (1) and (2) can now be applied to determine the water surface velocity and line-of-sight distance to the water surface. The line-of-sight distance to the water surface can be converted to a water depth measurement, *D*, via

(3) D=H−d sinθ

where *H* is the perpendicular distance from the radar sensor to the channel bed, which should be measured manually during the installation of the sensor.

#### 2.1.1. Electrical and Physical Overview

**Figure 1:**
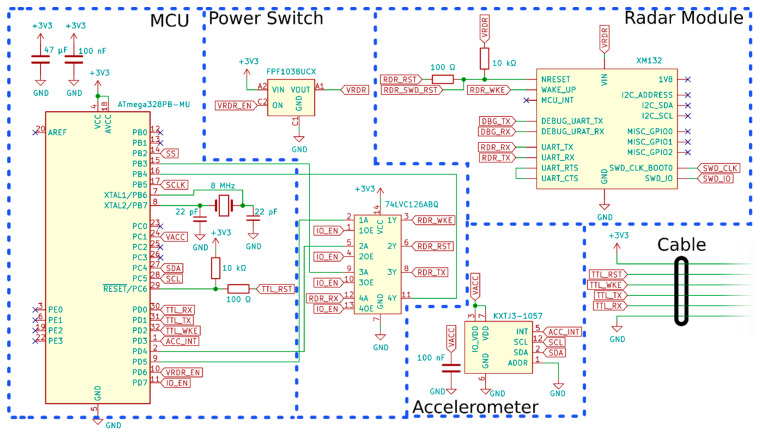
Electrical schematic for the sensor. The blue dashed line separates the functional components of the schematic. For a complete set of design files, please view the supplementary materials available with this article.

A schematic of the sensor is provided in Figure 1. It is separated into functional components. The MCU, an ATmega328PB, controls the other components of the printed circuit board (PCB) and handles the interface with the output. The ATmega328PB was chosen due to its ease of use and familiarity within sensing and datalogging applications [41]. A KXTJ3-1057 accelerometer measures the local acceleration of gravity to determine the orientation of the sensor. A power switch was incorporated in order to minimise the idle current draw of the sensor by turning components on and off as needed. In addition to handling the generation and detection of the radar signal, the radar module also has a custom developed processing algorithm to extract the depth and velocity.

**Figure 2:**
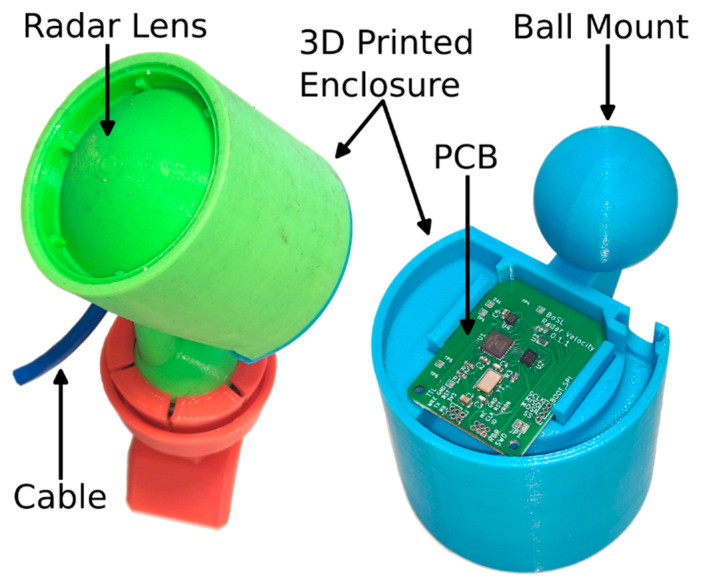
Picture of radar sensor labelling key features. Left: front view. Right: back view. The waterproofing back cover and cable are not shown in the back view so that the internal part of the sensor can be seen.

The sensor operates off a 3.3 V supply and communicates via a universal asynchronous receiver-transmitter (UART). A CAT5 cable carries this power and connectivity from a datalogger. The sensor body measures 60 mm in diameter and 60 mm in height and is entirely 3D-printed from polylactic acid (PLA) plastic. A picture of the sensor is provided in Figure 2. On the back of the sensor, there is a 15 mm radius ball mount which can be used to quickly attach the sensor during installations while still allowing for easy rotation of the sensor to the correct orientation. The front of the sensor houses a 3D-printed radar lens, which significantly boots the radar sensitivity. The back of the sensor is filled with a potting compound for waterproofing; however, there is an air cavity between the radar module and the lens required for radar operation. This cavity is waterproofed such that no water can enter it. The total cost of the sensor was less than USD 50 for the small production run of sensors for this study. A larger production run of a few hundred sensors would likely result in a lower per-unit cost.

#### 2.1.2. Radar Lens

Natively, the beam angle from the radar module is quite large, at a beam width of between 40° and 80° [42]. This is unideal for water measurement for several reasons: (a) a large beam width means that the radar power is dispersed over a wide area, hence only a small fraction is reflected back to the sensor; (b) narrow and distant water flows may only occupy a small fraction of the beam width, and hence not utilise the full output of the radar sensor; and (c) if the spot size of the radar beam on the water surface is too large, other objects present, such as pit walls or vegetation, may enter the radar beam and affect the measurement. For these reasons, it would be ideal to focus the beam width to a smaller angle. Recently, much work has been performed on developing radar quasioptics using low-cost additive manufacturing techniques such as fused deposition modeling (FDM) 3D printing [43,44,45,46,47]. Here we apply the advances of these previous works and incorporate such a 3D-printed radar lens to focus the radar beam. Many of these works have experimented with gradient refractive index to achieve a variable index of refraction. This work did not pursue this technology due to possibility of water ingress into metamaterials.

**Figure 3:**
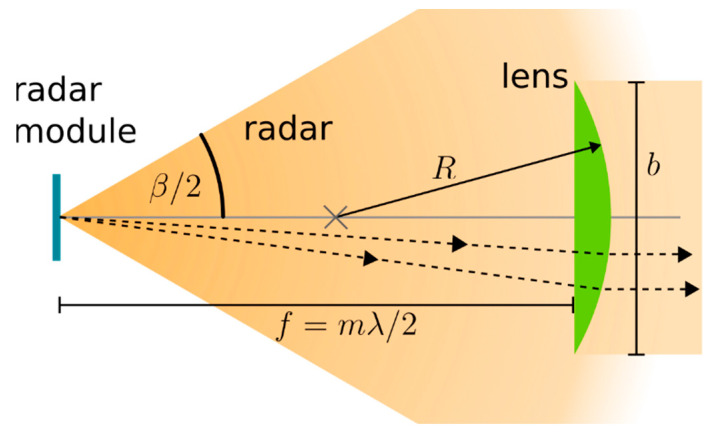
An optical diagram displaying the function of the radar lens. The falloff of radar intensity is illustrated in the orange background. Particular radar rays are displayed as dashed lines. Note how the radar lens focuses the rays from the radar module into a parallel beam reducing the intensity falloff with distance. *b* is the diameter of the lens, *R* the radius of curvature, and *f* the focal length. m is an arbitrary integer. *β* is the beam width angle of the radar emission. Paths of the rays through the lens are reversed for incoming radar signals.

As the radar lens is 3D-printed with the case out of common PLA plastic, it is very inexpensive to produce and easily adjustable to the optimal lens shape. At the same time, the addition of the radar lens brings a 21 dB gain to the collected radar power when compared with the absence of any radar lens. A diagram of how the radar lens works is shown in Figure 3. The radar lens is positioned in the case such that it focuses parallel incoming radar rays to the radar module located at the focal point of the lens.

Several lens designs and parameters were tested for optimal gain before the final design was settled on. The PLA plastic used for the manufacturing was measured to have a refractive index at 60 GHz of 1.8 ± 0.2. For the spherical planoconvex lens used here, the following is a result of geometric optics:

(4) $$\frac{1}{f}=\frac{n_{\mathrm{lens}}-n_{0}}{n_{0}}\frac{1}{R}$$

where *f* is the focal length of the lens, *n_lens_* is the refractive index of the lens material, *n*_0_ is the refractive index of the surrounding medium, and *R* is the radius of curvature of the lens. As long as the dimensions of the lens remain much larger than the wavelength, geometric optics can be used for analysis [48]. For a given plastic refractive index, this formula can be used to determine the radius of curvature to give optimal gain to the radar system. A further constraint of the focal length is that it should be a multiple of *λ*/2 to minimise the destructive interference of the radar signal reflecting between the radar module and the lens surface. Here, a focal length of *f* = 38 mm was chosen with an *R* = 30 mm radius of curvature. The diameter of the radar lens was made as large as is practical at *b* = 49 mm. This decreases diffractive effects and increases the proportion of the incoming radar signal collected, hence further increasing the receiver gain.

### 2.2. Data Processing

Data processing algorithms are needed to both determine the orientation of the sensor from the accelerometer readings and process the raw radar data collected to a final depth and velocity measurement. For determination of the orientation, the onboard accelerometer measures the acceleration of gravity in the local coordinates of the accelerometer. The accelerometer is mounted on the PCB such that the z-coordinate is parallel to the radar beam. Therefore, the angle between the radar beam and the horizontal is given by the following:

(5) $$\theta =\mathrm{atan}2\left(g_{z},\sqrt{g_{x}^{2}+g_{y}^{2}}\right)$$

where *g_x_*, *g_y_*, *g_z_* are, respectively, the *x*, *y*, *z* components of the acceleration as reported by the accelerometer, and *atan*2 is the two-argument arctangent function.

**Figure 4:**
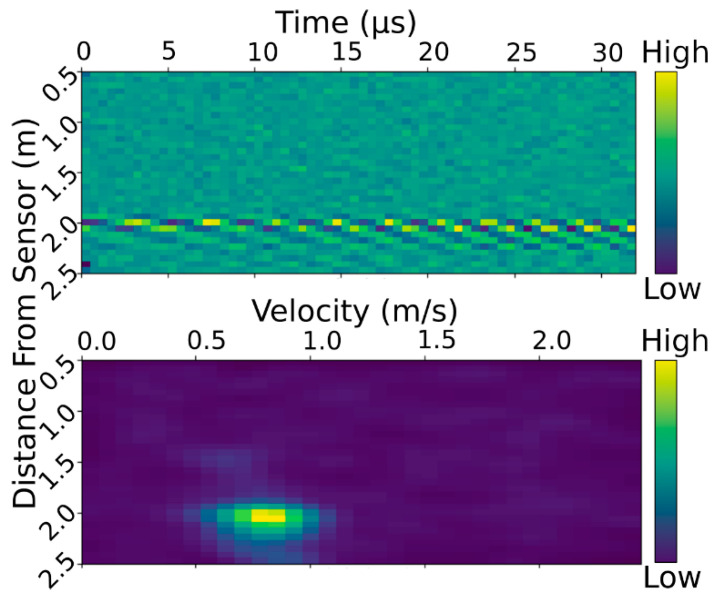
Typical radar scan data array. Top: raw reflected signal strength data returned by the radar module. Each bin represents the reflected signal strength from an object at the given distance from the sensor and at the given time. Signal strength is indicated by the colour scale. Bottom: radar after FFT processing on each data row. Each bin now represents the amplitude of the component frequency in the signal. The frequency scale is mapped to velocity via Equation (1). Colour indicates amplitude of frequency component in signal.

The determination of the water velocity and depth from the raw radar data is more involved. The radar module measures the signal strength reflected from the environment. These data are binned by the distance of the reflection from the sensor. These bins are 60 mm in length. The reflection data are measured 64 times at rate on the order of 1 kHz. For data processing, the reflection data are organized into a 2D array as shown in Figure 4 (top). In this array, the time of the measurement is given by the column and the distance bin by the rows. The start and end distances for the measurement of the reflected signal strength can be configured to suit the sensor’s installation. In this study, values of 240 mm and 2280 mm were used. To determine the water velocity, the Doppler velocity needs to be determined from the change in reflected signal strength over time. To accomplish this, a fast Fourier transform(FFT) is applied to each row of the array. This transforms the time axis into a frequency axis where the large values indicate the frequency has a large amplitude in the reflected signal (see bottom of Figure 4). Before the FFT is applied, the mean component of the signal is removed to detrend the data. This eliminates the contribution from stationary objects’ reflections which would otherwise obscure the dynamic signal from the water surface.

A single scan from the radar sensor completes all time samples within tens of microseconds. This is a very short time period compared with the random fluctuations of the water surface, so a single scan would be affected by this random fluctuation. Furthermore, the radar scan itself also has some uncertainty. To sample the water over a longer period of time and reduce uncertainty, the energy-preserving root mean square (RMS) average of six FFT processed scans, taken about one second apart, is calculated.

As per the theory behind radar velocity measurement [39], the signal does not have a single frequency peak, but rather a frequency spectrum. Therefore, to determine the Doppler frequency, an intelligent peak-finding algorithm is run on the RMS averaged array. The array is first preprocessed by applying a Gaussian blur with a kernel size of 1.5 bins. From here, the bin with the greatest amplitude is found; as this should occur in the bin corresponding to water surface – sensor distance, the analysis is then restricted to this row of the array. At this point, the value in the peak bin is taken as the ‘signal strength’ of the measurement. As the contrast between the peak and the background signal can be small, all bins with an amplitude less than one third of the maximum amplitude are set to zero. This eliminates most of the background signal, which can otherwise bias the readings to towards the centre of the measurement range. Finally, the amplitude-weighted average of the frequency bins is taken, which equates to a frequency that we consider to estimate the Doppler frequency Δ*f*. When combined with the angle measurement from the accelerometer *θ*, Equation (1) can be used to calculate the surface velocity.

To determine the distance to the water surface, a higher resolution mode on the radar module was used which provided the reflection amplitude in 2 mm increments but provided no time domain data. The bin with the greatest reflection amplitude was taken as the distance to the water surface.

### 2.3. Power Usage

To test the power usage of the sensor, a Multicomp Pro MP730026 ammeter was placed in series with the ground connection. The ammeter was then used to measure the sleep current of the sensor, which is the current while the sensor is not performing any measurements. This was found to be 100 nA. To measure the duration and charge needed for a single velocity and depth measurement, a Rigol DS1054Z oscilloscope was connected across the ammeter. As the shunt resistance of the ammeter was 4 Ω, this allowed for high time-resolution measurement of current consumption. A single measurement (which, as described above, is the average of 6 individual scans) was found to take 7.02 s and require 0.0742 mAh of charge. This results in an average current of 38.1 mA for a single measurement. The peak current could also be measured to be 64 mA. For a typical logging rate of 10 measurements per hour, the average current draw of the sensor is 740 μA. This provides 196 days of operation on a typical 3500 mAh single 18,650 Li-ion cell. For a logging rate of 1 measurement per hour, over 5 years of operation could be optimally achieved. This low power consumption supports the use case of the sensor in battery-operated installations with infrequent maintenance.

### 2.4. Lab Characterisation

**Figure 5:**
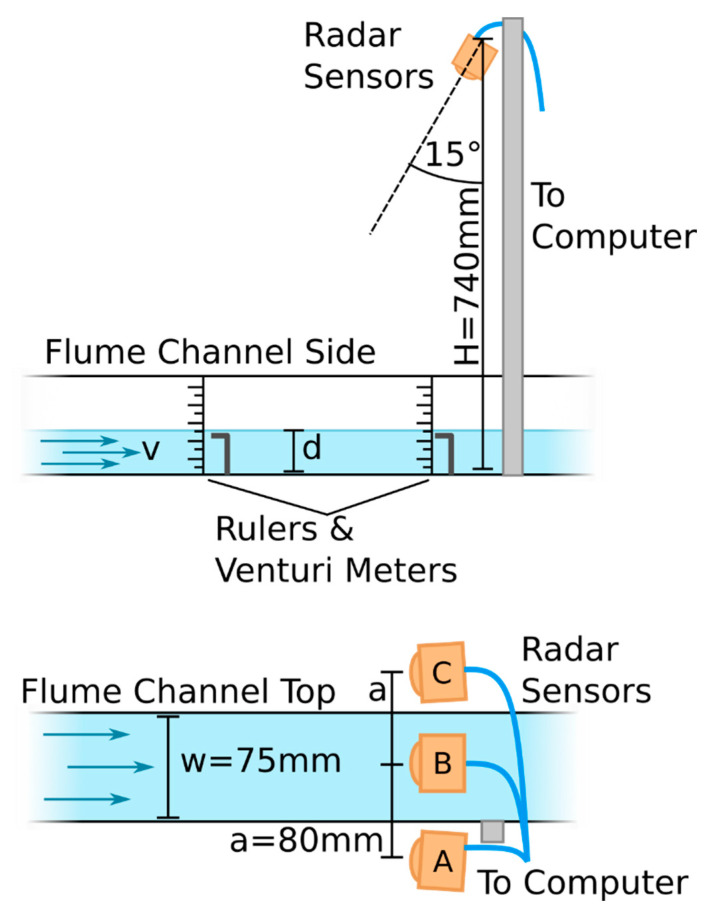
Diagram of setup for lab characterisation testing. The positions of radar sensors A, B, and C are shown. Top: side view, bottom: top view.

To gain an understanding of the performance of the sensor and its response to velocity and depth, a laboratory characterisation experiment was conducted. A flume was used to create conditions of varying depth and velocity (Figure 5, top). The water velocity and depth were adjusted by adjusting the flowrate and height of weirs in the flume. Due to the hydraulics of the flume, the data above a velocity of 0.5 m/s and below 25 mm of depth displayed supercritical flow as opposed to the subcritical flow when these conditions were not met. The ‘true’ depth was measured by using two sets of level indicators on either side of the section of water measured by the radar sensor. The average of these two measurements was taken to be ‘true’ water depth. The disagreement between the two level indicators never exceeded 2 mm, so the flow in the region measured by the radar sensor was relatively level. The flow was also observed to be in steady state. The ‘true’ velocity of the water in the flow was measured using a pair of venturi meters positioned in line with the two level indicators. Again, the average of the two venturi meters was taken to be the true velocity. As the velocity of the water is not uniform across the cross section of the flow, the venturi meters were positioned close to the surface to obtain a best estimate for the water surface velocity. This was performed because the Doppler radar measurement technique used by the sensor measures the velocity of the water surface.

Three radar sensors (A, B, and C) were mounted a distance of H = 740 mm above the channel bed and pointing at an angle of 74° from the horizontal. Sensor B was placed along the thalweg of the flow with sensors A and C offset by 60 mm to the left and right sides, respectively (Figure 5 bottom). Multiple sensors were used to triplicate the data and gain an understanding of the variance in performance between the sensors. The radar data were recorded from each of the three sensors onto a computer via UART. Each datapoint is the average of 12 consecutive measurements. The results presented in this work were analysed using the processing algorithm explained above.

### 2.5. Field Trial

**Figure 6:**
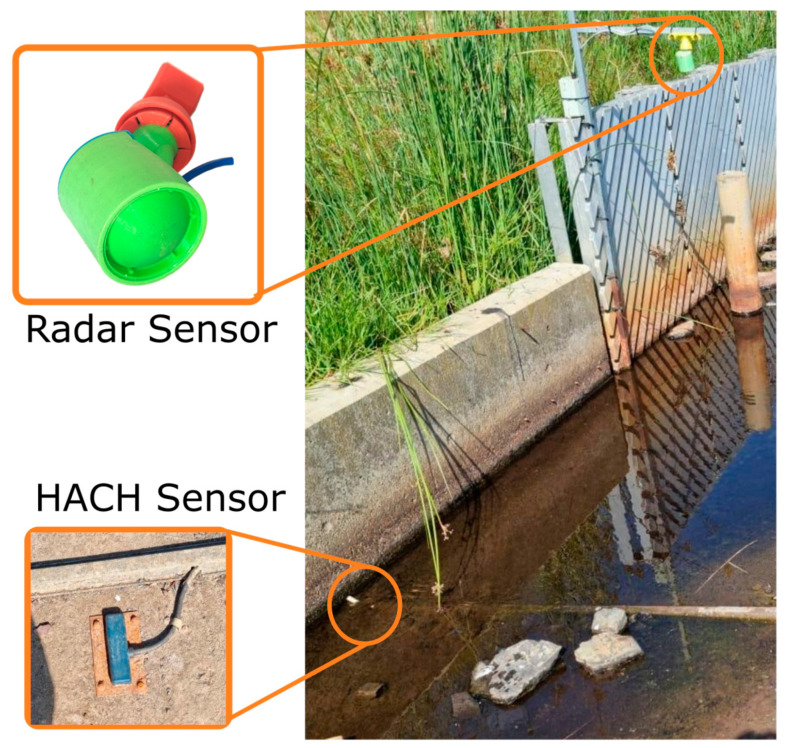
Photograph of site setup at Troup’s Creek. Note the location of the radar sensor in the top right of the image. The HACH sensor is located at the left end of the cable shroud, the deepest point of the channel. Both the HACH sensor and cable shroud are visible in the inset. The direction of water flow is forward in the image.

For the field trial, the sensor was deployed for 5 weeks at the inlet of Troup’s Creek Wetland in Melbourne, Australia. Over this period, the channel was mostly dry; however, two large flow events were captured. The radar sensor was connected to a datalogger which uploaded a measurement from the sensor every 10 min to a cloud server. The radar sensor was compared against a commercial HACH AV9000 Submerged Area Velocity Sensor [17], an in-water acoustic Doppler velocity and level sensor. The HACH probe was installed at the bed of the channel and was set to log locally at intervals of 1 min. A photograph of this setup is provided in Figure 6. For the field trial, it was initially assumed that the HACH sensor recorded the true velocity and depth of the channel. The validity of this assumption is questionable, as is discussed in the results. At certain times during the trial, the HACH sensor reported failed velocity and depth measurements, usually due to an obstruction in front of the sensor. These periods of time are identified by a high density of identical velocity readings from the HACH sensor and have been removed. The data were also filtered so that all of the following were true: (a) the radar sensor was installed within the recommended operating angle of between 70° and 87° (on some occasions, birds or humans would move the sensor, which would cause the sensor to be pointing at objects other than the water flow), (b) the radar depth was non-negative and the water surface was further than 350 mm from the sensor, and (c) the HACH depth was above 100 mm. This last condition was necessary as the cable shroud (Figure 6) around the HACH sensor cable was observed to create a small hydraulic jump at low depths, invalidating velocity readings. The installation height of the radar sensor was measured manually during installation and used to determine the water depth from the distance data. A HACH datapoint was associated with each radar sensor measurement by finding the temporally nearest HACH datapoint. Therefore, the maximum time difference between the HACH sensor measurement and the radar sensor measurement was ±1 min.

## 3. Results

### 3.1. Lab Characterisation

**Figure 7:**
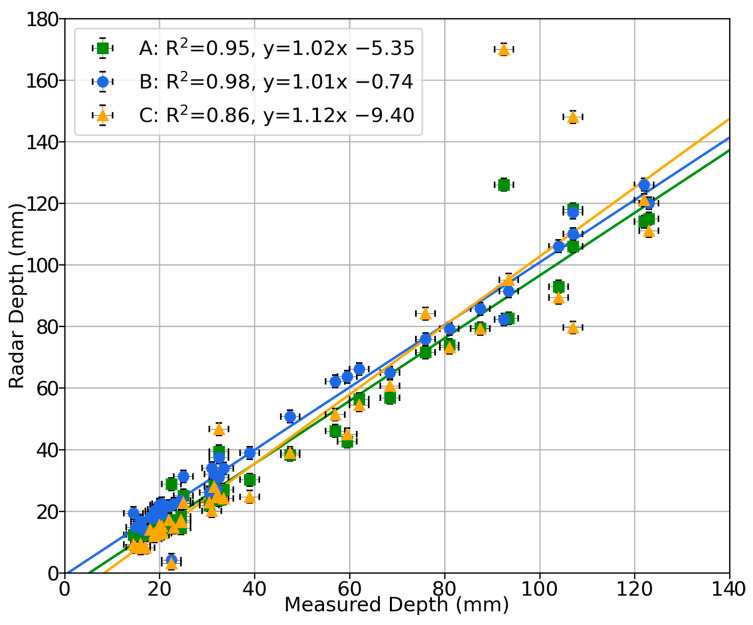
Correlation plot of measured depth against sensor depth from laboratory characterisation. The results from sensors A, B, and C along with a linear trendline for each are shown. The equations of the linear trendlines and R^2^ coefficients are displayed in the plot legend.

The performance of the sensor’s depth measurement is shown in Figure 7. A strong linear trend is present in the data, with R^2^ coefficients greater than 0.86, and the probability of the null hypothesis that the gradient is zero is *p* < 0.0001. For sensors A and B, the depth response differs from unity by less than 2% while the depth offset is less than 6 mm. Sensor C does not have as ideal a response, however, with a gradient error of 12% and a 9 mm offset. It is possible that some of this uncertainty in the depth measurement is due to the placement of sensors A and C off the centreline of the flume, as the offset slightly affects the equations needed to recover the depth and velocity, and this offset may also affect the sensor’s line of sight to the water. The root mean square (RMS) errors of each measurement from their linear fit are 17%, 16%, and 24% for sensors A, B, and C, respectively. Due to constraints in the laboratory flume, only a small depth range of 15 mm to 125 mm could be tested; however, on this range it is seen that the sensor produces an accurate response to changes in the water depth.

**Figure 8:**
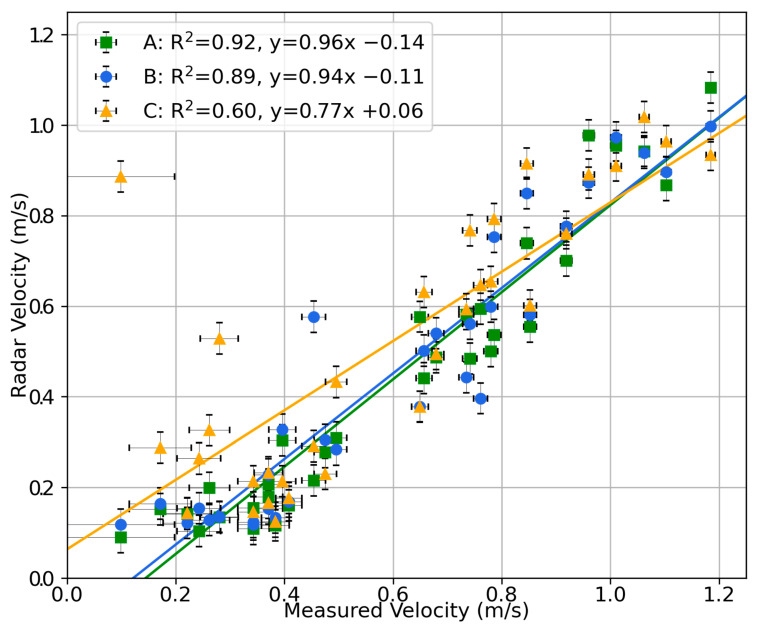
Correlation plot of measured velocity against sensor velocity from laboratory characterisation. The results from sensors A, B, and C along with a linear trendline for each are shown. The equations of the linear trendlines and R^2^ coefficients are displayed in the plot legend.

The performance of the sensor’s velocity measurements is shown in Figure 8. Again, a strong linear trend is present in the data. All of the linear trendlines had a probability of the null hypothesis for a gradient of zero of *p* < 0.0001. The velocity data also show that sensor C does not perform as strongly as sensors A or B; this could be due to either unideal positioning of sensor C or a defect in sensor C’s assembly. Considering only sensors A and B, the linear correlation coefficient is greater than 0.89 and the gradient is within 6% of unity. The offset is less than 0.14 m/s. It is seen, however, that below 0.3 m/s, the response of the radar velocity to changes in measured velocity is limited. This represents that the sensor has difficulty in resolving low velocities; however, this is somewhat unsurprising as it is a common issue faced by radar-based velocity sensing [19]. Due to this differing behaviour past 0.3 m/s, considering only the data above 0.3 m/s, sensors A, B, and C have RMS errors from their linear fit of 20%, 28%, and 30%, respectively. This figure gives an indication of the point-to-point error expected for a velocity measurement and is due to both a systematic and random error component. In field applications, where the measurement interval is much shorter than the characteristic timescale of velocity changes, averaging neighbouring measurements helps reduce the random component of this error; however, this is not able to address the systematic component. These data demonstrate the capability of the sensor to effectively measure the surface water velocity up to at least the 1.2 m/s tested in this experiment. While the sensor was installed quite close to the water surface (740 mm from the flume bed), the radar signal was also attenuated by the 10 mm acrylic top of the flume, which the radar signal had to pass through. These data demonstrate some capacity for the radar sensor to measure through certain small obstructions to a direct line of sight to the water surface. This suggests another application for the sensor, that of external and unintrusive measurement of open channel flow in small pipes. Together, these lab characterisation tests provide evidence for the sensor’s ability to measure both depth and velocity.

### 3.2. Field Trial

**Figure 9:**
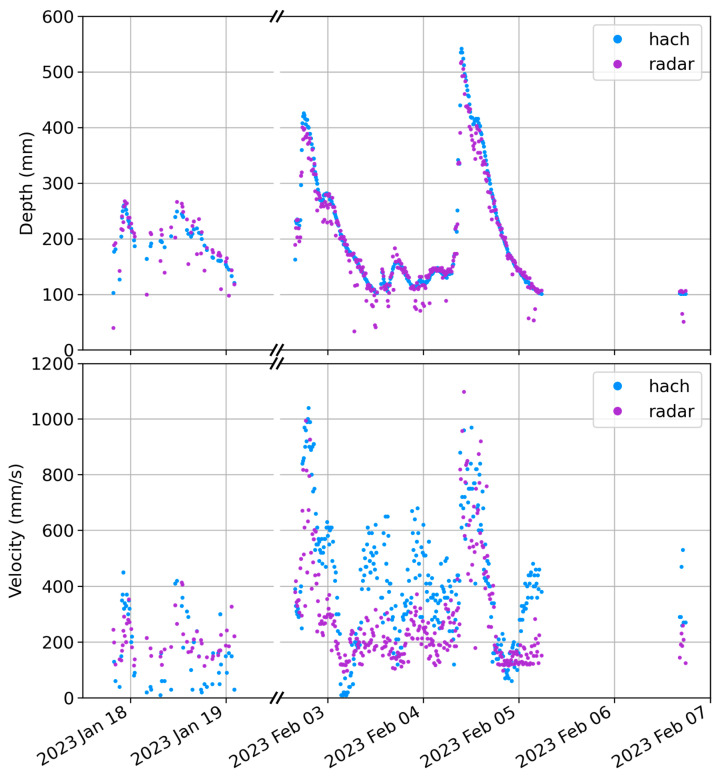
Plot of timeseries data from field trial. Top: depth measurement. Bottom: velocity measurements. Note that many of the depth data are overlapping in the HACH and radar data series.

The timeseries data recorded over the duration of the field trial are displayed in Figure 9. The depth data (Figure 9, top) show that the new radar sensor’s depth measurements agree with the HACH measurements, with most data mostly superimposed on each other. It is seen, however, that there are a few sporadic depth measurements where isolated depth measurements from the radar sensor are lower than neighbouring measurements; again, this is most prevalent at low depths. While it is not certain what causes these sporadic points, they may be due to the effect of background clutter (ground, plants, etc.) [23]. Given their sparsity and distinctness from the rest of the depth data series, it is believed that a simple filtering algorithm could be applied to these data to reduce any significant jumps in the dataset. Furthermore, we believe the sensor data could be more thoroughly explored to provide uncertainty estimates of each measurement, either by a) using the variability in the six individual scans or b) using the strength information as a quality scoring system.

**Figure 10:**
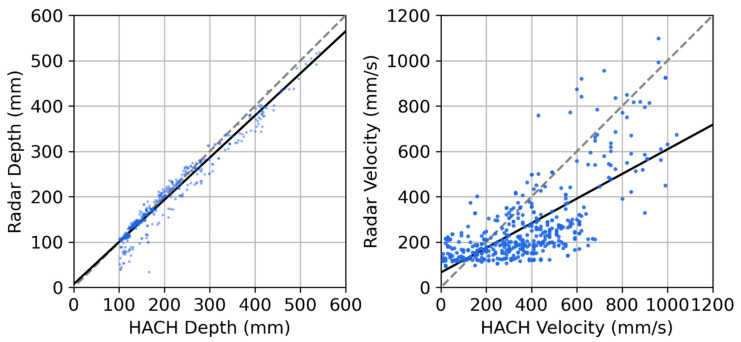
Left: depth correlation. Right: velocity correlation. Black line: line of best fit. Grey line: 1:1 correlation line. The line of best fit for the depth correlation is y = 0.93x − 7 mm with an R^2^ of 0.88. The line of best fit for the velocity correlation is y = 0.54x + 66 mm/s with an R^2^ of 0.48. Note that a few datapoints extended beyond the axis and have been clipped.

Figure 10 shows a strong linear correlation between the depth values from the radar and HACH sensors, with a line of best fit having an R^2^ of 0.88, a gradient of 0.93, and an intercept of 7 mm. The probability of the null hypothesis that the gradient is zero for the regression is *p* < 0.0001, reconfirming that precise absolute depth measurements are possible from this radar sensor, even with only a single measurement of the installation height of the sensor. The mean absolute error of the sensor is estimated to be 15%, and this is greater than what most existing commercial sensors promise [15,27,49] (which are often accurate to a few percent or mm). However, these are often provided for ideal laboratory conditions and others do report extremely high uncertainties (e.g., >30%) when these commercial sensors are used in field conditions, especially during times of low water depths [50]. These quoted uncertainties serve as a lower bound for the performance, as (a) the sporadically low points have not been filtered in this analysis; (b) the analysis assumes that the HACH sensor provides the true water depth when in reality it has an uncertainty of at least 2%, located upstream of the radar sensor, and its datasets were only synchronized up to a ±1 min error; and (c) the radar sensor can be more susceptible to passing debris and variance in water level due to turbulence and surface waves, whereas these changes would not be picked up by the in-water HACH sensor.

For the velocity data, there are again similar trends between the two sensors, demonstrating the potential of the radar sensor to estimate water velocities (Figure 9, bottom). A linear trend is also seen between the HACH and the low-cost radar sensor (Figure 10, right), with an R^2^ of 0.48, a gradient of 0.54, and an intercept of 66 mm/s. The radar velocity data have a mean absolute error (MAE) from their trendline of 36%. The probability of the null hypothesis that the gradient is zero is *p* < 0.0001, implying the fit is significant. This general agreement is an important finding, demonstrating the ability of the low-cost, low-power, noncontact sensor to detect water velocities and providing an opportunity for collecting highly spatially distributed datasets of water movements throughout complex catchments.

The deviations observed between the radar and the HACH sensors were, however, greater for the velocity measurements than for the depth measurements. This was expected, as velocity measurements are far more complex and always have higher uncertainties than depth measurements [50]. The fact that open water flows have varying velocities throughout their cross section makes comparing one sensor with another even more challenging as deviations between sensors deployed in the field are further exacerbated due to the following: (a) the technique used for estimating velocity (e.g., water column velocity measurements vs. surface water velocities) and (b) the installation characteristics of the sensor (e.g., wall effects causing the sensor’s measurements to be less representative of the actual water’s velocity).

We believe that these factors resulted in the deviations we observe in Figure 9 (bottom) and Figure 10 (right). First, while both the HACH sensor and the radar sensor rely on the same fundamental physics (Doppler shift), they are measuring different parts of the water velocity profile, with one being out of the water column and the other being inside the water. Indeed, prior research demonstrates that the velocity can significantly change within a cross section of the water flow [51], and that this profile changes depending on the flow conditions. Under many conditions, the surface velocity can be 20% lower than the average cross-sectional velocity [51], especially if the flow is now measured along the thalweg. This likely contributes to the 0.54 gradient of the line when comparing the HACH with the radar sensor. Importantly, the differences between surface water velocities and average in-water velocities are often the greatest at low flow velocities and low depths, which are where the radar sensor and the HACH sensors deviate the most. Furthermore, the sensitivity of the in-water sensor is likely to be higher than that of the radar sensor, which relies on remote sensing style measurements, likely contributing to the plateau observed in the correlation plot (i.e., no variation in radar sensor response up to 300 mm/s) and the intercept of 66 mm/s.

The installation characteristics of the two sensors also need to be considered when interpreting Figure 9 and Figure 10. Indeed, due to vandalism, the HACH sensor was installed with a metal shroud to protect its cabling, as is visible in Figure 6. This was observed to act as a small hydraulic structure, particularly impacting low flow depths and hence low water velocities, and was assumed to increase in-water velocities by impacting acoustic signals that travel through the flow over the structure. This would have contributed to the large deviations observed in the velocities from the two sensors, especially during times of low water depths. In fact, the largest deviations observed in Figure 9 (bottom) occurred during the times of the lowest water depths (3 and 4 February), suggesting that this structure could also have contributed to these results.

To explore the inherent difficulties in comparing two sensors and to begin to understand the performance of the radar sensor in more detail, we compared two identical HACH AV sensors installed in the same cross section. The second in-water HACH sensor was installed 1 m to the right of the original HACH sensor, as seen in Figure 6. The R^2^ between the two identical HACH sensors was 0.56 and the right HACH sensor measured 62% higher on average. These values are not dissimilar to the values we reported above for the comparison between the original HACH sensor and the radar sensor. The disagreement between these two sensors installed in similar conditions is indicative of the challenge of in-field velocity measurement and the typical degradation of performance in field vs. lab tests. This difference occurs despite the HACH sensors being state-of-the-art commercial sensors.

In summary, we provide the evidence above to demonstrate that the radar sensor is capable of estimating water velocities (i.e., R^2^ = 0.48 when compared with a commercial sensor). However, it is noted that this sensor is measuring surface water velocities, which are related, but not equivalent, to the cross-sectional average velocity (hence resulting in a slope of the line of best fit of 0.54 when compared with the instream sensor). We further note that although installation characteristics limit our comparison between the HACH and the radar sensor, this field dataset, along with the extensive laboratory datasets, implies that the uncertainties in the sensor for velocities less than 300 mm/s are significant. While the poor performance of the radar sensor below 300 mm/s precludes the application of this radar sensor in measuring velocity in slow water bodies, it is less of a limitation for stormwater, wastewater, and rivers, in which the events of interest tend to have velocities well in excess of 300 mm/s. Future research could explore how radar lenses with greater gain or narrower cone angles could help to reduce these uncertainties.

### 3.3. Comparison with the Literature

While a strict comparison of the performance of this sensor with others available on the market is difficult due to the limited information provided in these publications, we attempt to perform a qualitative comparison with the literature, as summarised below and in Table 1. An implementation of Doppler radar using a custom-designed integrated circuit obtained an uncertainty of 8% in ideal conditions at the single velocity it was tested at of 1.2 m/s [35]. The 8% figure is likely an underestimate of the sensor’s mean absolute error. For our sensor, while our error was 36% in field conditions and between 20% and 30% for the lab tests, this was over the entire range of the sensor. The low-cost, low-power in-water sensor proposed in [22] has RMS errors ranging from 17% to 43% between 200 mm/s and 1200 mm/s in long-term field testing. This performance is comparable with that of the radar sensor here. Image velocimetry from unmanned aerial drones (UAV) was attempted in [31]; in flow velocities of up to 200 mm/s, the average difference from a reference ADCP sensor was 50 mm/s. This equates to a minimum average error of 25%. A second image velocimetry from the UAV study found mean absolute errors of between 60 mm/s and 140 mm/s in flows of up to 1100 mm/s [28]. These values are 5% and 13% of the peak flowrate, respectively. A low-cost, low-power radar Doppler sensor utilising a custom radar board achieved mean errors of −3% and −11% at the two flow velocities tested in a field-day test [34]. Furthermore, [29] studied the accuracy of the commercial Decatur Surface Velocity Radar [52] at a range of sites and found that its surface velocity error ranged from 7% to 25%. From this comparison with the current literature (Table 1), our proposed sensor, while having a slightly higher average uncertainty, is near the range of the uncertainties of other sensors. The radar sensor also has the advantages of being out-of-water, low-cost, low-power, and capable of simultaneous depth measurement.

## 4. Conclusions

The radar velocity and depth sensor designed and verified here was tested and found to successfully measure depth and velocity in both laboratory and field trials. The depth response was found to be highly linear, with the linear regression having a gradient differing only by at most 6% from unity and offsets of less than 7 mm in both lab and field tests. The average error in the surface velocity measurement was found to be less than 30% in lab conditions and 36% in real-world conditions from the mean velocity. The velocity uncertainty is greatest in slow-moving water of velocities of less than 300 mm/s. However, this limitation does not significantly inhibit stormwater, wastewater, and river monitoring applications as velocities of interest tend to be well above 300 mm/s. While the velocity error is larger than those of the best available sensors, the sensor proposed here has the advantage of being the first to compete with such sensors at a low cost (USD <50). Its low power consumption and out-of-water installation reduce maintenance frequency and cost. This supports low-cost long-term deployment, enabling wide arrays of these sensors to be installed to collect data over a large area with high spatial resolution. These deployment schemes have been previously prohibited by cost. The design of the sensor is made freely available and utilises off-the-shelf components. The use of 3D printing technology for the casing allowed a novel sensitivity-enhancing radar lens to be included at minimal cost. The sensor is also interfaceable with a wide array of dataloggers, and has an onboard accelerometer for automatic inclination detection.

## Figures and Tables

**Table 1:** Comparison of our work with existing velocity sensors. While a cost estimate is not available for all works, as low cost was not a target for these works, their costs are likely to be significantly higher than USD 50.

Ref.	Use	State	Type	Cost	Velocity Uncertainty	Uncertainty Method
This work	Out-of-water	Field-ready	60 GHz radar	<50 USD	36%	MAE (field)
[35]	Out-of-water	Prototype	24 GHz radar	n.a.	8% @ 1.2 m/s	Spot difference (field)
[22]	In-water	Field-ready	Acoustic Doppler	<50 USD	43%	RMS (field)
[31]	UAV	Field-ready	Image velocimetry	n.a.	25%	MAE (field)
[28]	UAV	Field-ready	Image velocimetry	n.a.	13%	MAE (field)
[34]	Out-of-water	Prototype	24 GHz radar	<50 USD *	−3% @ 1.4 m/s −11% @ 1.2 m/s	Mean error (field)
[29,52]	Out-of-water (handheld)	Field-ready	24 GHz radar	1500 USD	25%	Local error (field)

* Hypothesised cost if mass-produced in silicon system-on-chip.

## Data Availability

The data presented in this study are available in the supplementary materials.

## Supplementary Materials

The following supporting information can be downloaded at: https://www.mdpi.com/article/10.3390/s23146314/s1. Please find the complete design files necessary to modify and build the sensors, along with the data used in the analysis of this study, in the supplementary materials.

## References

[1] Lim S.-R., Suh S., Kim J.-H., Park H.S. (2010). Urban Water Infrastructure Optimization to Reduce Environmental Impacts and Costs. J. Environ. Manag..

[2] Fallah Shorshani M., Bonhomme C., Petrucci G., André M., Seigneur C. (2014). Road Traffic Impact on Urban Water Quality: A Step towards Integrated Traffic, Air and Stormwater Modelling. Environ. Sci. Pollut. Res..

[3] Herrera M., Torgo L., Izquierdo J., Pérez-García R. (2010). Predictive Models for Forecasting Hourly Urban Water Demand. J. Hydrol..

[4] Brzezińska A., Zawilski M., Sakson G. (2016). Assessment of Pollutant Load Emission from Combined Sewer Overflows Based on the Online Monitoring. Environ. Monit. Assess..

[5] Passerat J., Ouattara N.K., Mouchel J.-M., Rocher V., Servais P. (2011). Impact of an Intense Combined Sewer Overflow Event on the Microbiological Water Quality of the Seine River. Water Res..

[6] Hauet A.C. (2020). Uncertainty of Discharge Measurement Methods: A Literature Review.

[7] On the Derivation of Flow Rating Curves in Data-Scarce Environments|Elsevier Enhanced Reader. https://reader.elsevier.com/reader/sd/pii/S0022169418303111?token=E7BF89DB02F822C933091A38091751208C04D135804463AABDF46E53F598519B735CAC22AECF982A37CAD1857634B495&originRegion=us-east-1&originCreation=20220916025705.

[8] Baldassarre G.D., Montanari A. (2009). Uncertainty in River Discharge Observations: A Quantitative Analysis. Hydrol. Earth Syst. Sci..

[9] Moramarco T., Barbetta S., Tarpanelli A. (2017). From Surface Flow Velocity Measurements to Discharge Assessment by the Entropy Theory. Water.

[10] Gore J.A., Banning J., Hauer F.R., Lamberti G.A. (2017). Chapter 3—Discharge Measurements and Streamflow Analysis. Methods in Stream Ecology.

[11] Chen Y.-C., Hsu Y.-C., Zai E.O. (2022). Streamflow Measurement Using Mean Surface Velocity. Water.

[12] Vyas J.K., Perumal M., Moramarco T. (2020). Discharge Estimation Using Tsallis and Shannon Entropy Theory in Natural Channels. Water.

[13] Bonhomme C., Petrucci G. (2017). Should We Trust Build-up/Wash-off Water Quality Models at the Scale of Urban Catchments?. Water Res..

[14] Shi B., Bach P.M., Lintern A., Zhang K., Coleman R.A., Metzeling L., McCarthy D.T., Deletic A. (2019). Understanding Spatiotemporal Variability of In-Stream Water Quality in Urban Environments—A Case Study of Melbourne, Australia. J. Environ. Manag..

[15] Kerkez B., Gruden C., Lewis M., Montestruque L., Quigley M., Wong B., Bedig A., Kertesz R., Braun T., Cadwalader O. (2016). Smarter Stormwater Systems. Environ. Sci. Technol..

[16] Fulton J.W., Anderson I.E., Chiu C.-L., Sommer W., Adams J.D., Moramarco T., Bjerklie D.M., Fulford J.M., Sloan J.L., Best H.R. (2020). QCam: SUAS-Based Doppler Radar for Measuring River Discharge. Remote Sens..

[17] HACH Submerged Area/Velocity Sensor and AV9000. https://au.hach.com/asset-get.download.jsa?id=8289724802.

[18] Ahmed U., Mumtaz R., Anwar H., Mumtaz S., Qamar A.M. (2019). Water Quality Monitoring: From Conventional to Emerging Technologies. Water Supply.

[19] Fulton J.W., Mason C.A., Eggleston J.R., Nicotra M.J., Chiu C.-L., Henneberg M.F., Best H.R., Cederberg J.R., Holnbeck S.R., Lotspeich R.R. (2020). Near-Field Remote Sensing of Surface Velocity and River Discharge Using Radars and the Probability Concept at 10 U.S. Geological Survey Streamgages. Remote Sens..

[20] Zhu Q., Cherqui F., Bertrand-Krajewski J.-L. (2023). End-User Perspective of Low-Cost Sensors for Urban Stormwater Monitoring: A Review. Water Sci. Technol..

[21] Ristolainen A., Tuhtan J.A., Kruusmaa M. (2019). Continuous, Near-Bed Current Velocity Estimation Using Pressure and Inertial Sensing. IEEE Sens. J..

[22] Catsamas S., Shi B., Deletic B., Wang M., McCarthy D.T. (2022). A Low-Cost, Low-Power Water Velocity Sensor Utilizing Acoustic Doppler Measurement. Sensors.

[23] Huang Y., Chen H., Liu B., Huang K., Wu Z., Yan K. (2023). Radar Technology for River Flow Monitoring: Assessment of the Current Status and Future Challenges. Water.

[24] Shi B., Catsamas S., Deletic B., Wang M., Bach P.M., Lintern A., Deletic A., McCarthy D.T. (2022). Illicit Discharge Detection in Stormwater Drains Using an Arduino-Based Low-Cost Sensor Network. Water Sci. Technol..

[25] Prafanto A., Budiman E. A Water Level Detection: IoT Platform Based on Wireless Sensor Network. Proceedings of the 2018 2nd East Indonesia Conference on Computer and Information Technology (EIConCIT).

[26] Mohammed S.L., Al-Naji A., Farjo M.M., Chahl J. (2019). Highly Accurate Water Level Measurement System Using a Microcontroller and an Ultrasonic Sensor. IOP Conf. Ser. Mater. Sci. Eng..

[27] Wang G., Gu C., Rice J., Inoue T., Li C. Highly Accurate Noncontact Water Level Monitoring Using Continuous-Wave Doppler Radar. Proceedings of the 2013 IEEE Topical Conference on Wireless Sensors and Sensor Networks (WiSNet).

[28] Bandini F., Frías M.C., Liu J., Simkus K., Karagkiolidou S., Bauer-Gottwein P. (2022). Challenges with Regard to Unmanned Aerial Systems (UASs) Measurement of River Surface Velocity Using Doppler Radar. Remote Sens..

[29] Welber M., Le Coz J., Laronne J.B., Zolezzi G., Zamler D., Dramais G., Hauet A., Salvaro M. (2016). Field Assessment of Noncontact Stream Gauging Using Portable Surface Velocity Radars (SVR). Water Resour. Res..

[30] Thumser P., Haas C., Tuhtan J.A., Fuentes-Pérez J.F., Toming G. (2017). RAPTOR-UAV: Real-Time Particle Tracking in Rivers Using an Unmanned Aerial Vehicle. Earth Surf. Process. Landf..

[31] Pearce S., Ljubičić R., Peña-Haro S., Perks M., Tauro F., Pizarro A., Dal Sasso S.F., Strelnikova D., Grimaldi S., Maddock I. (2020). An Evaluation of Image Velocimetry Techniques under Low Flow Conditions and High Seeding Densities Using Unmanned Aerial Systems. Remote Sens..

[32] Koutalakis P., Tzoraki O., Zaimes G. (2019). UAVs for Hydrologic Scopes: Application of a Low-Cost UAV to Estimate Surface Water Velocity by Using Three Different Image-Based Methods. Drones.

[33] Watanabe K., Fujita I., Iguchi M., Hasegawa M. (2021). Improving Accuracy and Robustness of Space-Time Image Velocimetry (STIV) with Deep Learning. Water.

[34] Alimenti F., Bonafoni S., Gallo E., Palazzi V., Vincenti Gatti R., Mezzanotte P., Roselli L., Zito D., Barbetta S., Corradini C. (2020). Noncontact Measurement of River Surface Velocity and Discharge Estimation with a Low-Cost Doppler Radar Sensor. IEEE Trans. Geosci. Remote Sens..

[35] Lin Y.-S., Chiu S.-F., Chang C.-H. (2020). A 24 GHz Hydrology Radar System Capable of Wide-Range Surface Velocity Detection for Water Resource Management Applications. Microw. Opt. Technol. Lett..

[36] Fernandes D., de Góes R.E., Fabiani A.L.T., Barreto R.C., Kamikawachi R.C. (2022). Self-Temperature Compensated Water Level and Velocity Sensor Based on Fiber Bragg Gratings. IEEE Sens. J..

[37] Wang J.-N., Luo C.-Y. (2012). Long-Period Fiber Grating Sensors for the Measurement of Liquid Level and Fluid-Flow Velocity. Sensors.

[38] Acconeer XM132 Datasheet. https://developer.acconeer.com/download/xm132-datasheet-pdf/.

[39] Plant W.J., Keller W.C., Hayes K. (2005). Measurement of River Surface Currents with Coherent Microwave Systems. IEEE Trans. Geosci. Remote Sens..

[40] Costa J.E., Cheng R.T., Haeni F.P., Melcher N., Spicer K.R., Hayes E., Plant W., Hayes K., Teague C., Barrick D. (2006). Use of Radars to Monitor Stream Discharge by Noncontact Methods. Water Resour. Res..

[41] Bradley L.J., Wright N.G. (2020). Optimising SD Saving Events to Maximise Battery Lifetime for Arduino^TM^/Atmega328P Data Loggers. IEEE Access.

[42] A111 Datasheet.Pdf—Onehub. https://developer.acconeer.com/download/a111-datasheet-pdf/.

[43] Abedi H., Shaker G. Low-Cost 3D Printed Dielectric Hyperbolic Lens Antenna for Beam Focusing and Steering of a 79GHz MIMO Radar. Proceedings of the 2020 IEEE International Symposium on Antennas and Propagation and North American Radio Science Meeting.

[44] Friel R.J., Gerling-Gerdin M., Nilsson E., Andreasson B.P. (2019). 3D Printed Radar Lenses with Anti-Reflective Structures. Designs.

[45] Munina I., Grigoriev I., O’donnell G., Trimble D. (2023). A Review of 3D Printed Gradient Refractive Index Lens Antennas. IEEE Access.

[46] Hagström A.L., Vass L.A.M., Liu F., Gerling M., Karlsson P.-O., Nilsson E., Andreasson B.P. An Iterative Approach to Determine the Refractive Index of 3D Printed 60GHz PLA Lenses. Proceedings of the Loughborough Antennas & Propagation Conference (LAPC 2018).

[47] Paolella A.C., Fisher C.D., Corey C., Foster D., Silva-Saez D. (2018). 3-D Printed Millimeter-Wave Lens Systems at 39 GHz. IEEE Microw. Wirel. Compon. Lett..

[48] Fernandes C.A., Lima E.B., Costa J.R., Chen Z.N., Liu D., Nakano H., Qing X., Zwick T. (2016). Dielectric Lens Antennas. Handbook of Antenna Technologies.

[49] Shi B., Catsamas S., Kolotelo P., Wang M., Lintern A., Jovanovic D., Bach P.M., Deletic A., McCarthy D.T. (2021). A Low-Cost Water Depth and Electrical Conductivity Sensor for Detecting Inputs into Urban Stormwater Networks. Sensors.

[50] McCarthy D.T., Deletic A., Mitchell V.G., Fletcher T.D., Diaper C. (2008). Uncertainties in Stormwater *E. coli* Levels. Water Res..

[51] Maghrebi M.F., Rahimpour M. (2005). A Simple Model for Estimation of Dimensionless Isovel Contours in Open Channels. Flow Meas. Instrum..

[52] Decatur Europe—Surface Velocity Radar|Hand-Held SVR Radar for Water Flow Measurement. https://www.decatureurope.com/en/products/surface-velocity-flow.

