# Supplementary material for: A Low-Cost Radar-Based IoT Sensor for Noncontact Measurements of Water Surface Velocity and Depth
Source: Sensors (Basel). 2023 Jul 11;23(14):6314. doi: 10.3390/s23146314 (PMC10385264; doi:10.3390/s23146314)
Supplement: Supplementary file 1 [file sensors-23-06314-s001.zip › supmat/design_files/assembly_instructions.pdf]

Assembling the Radar Sensor

This page will be dedicated to instructions of how to assemble the radar sensor. A video tutorial can be found at the end of this page.

Contents

Firmware
- Tips and Tricks

3D Printing
- Lens
- Backplate
- Body

Testing

Cabling

Waterproofing
- Backplate
- Lens

Video Tutorial

Firmware

To load the firmware onto the radar module please follow the following instructions.

The below image is of the back of the radar PCB. Looking from the back of the PCB, there are three sets of 6 pin connections. As labelled in the diagram red, green, and blue. The red connection is used to program and talk to the ATmega it is to be connected to by a bosl programmer probe. The green connection is a duplicate of the red connection however the pin pitch is greater. This is where the sensor cable is soldered. The blue connections is for programming the XM132. It is a serial wire debug interface designed to be connected to by a bosl programmer probe.

To begin to upload the firmware connect the red connection to a 3.3V serial port, connect the blue connection to a SWD debugger, such as the segger J-link.

1. To program the XM132, a special firmware for the ATmega need to first be programmed which allows this. This can be done via uploading the following sketch from the Arduino IDE: File:Radar Velocity STM resetremap.zip. For all uploading the to the ATmega please use the ATmega328PB board supplied in the BoSL Boards pack of the arduino board manager, (available here: https://monash-bosl.github.io/BoSL-IDE-Core/) For this use settings shown in the screenshot.

1. The radar firmware can then be uploaded onto the XM132 using the SWD debug probe. The target MCU is the STM32G071CB. The latest release can be found here https://github.com/Monash-BoSL/xm132-radar-firmware/releases/tag/v1.6.2 (use the .hex file)
2. The radar firmware can now be flashed onto the ATmega using similar settings to step 1. The radar firmware is available here:

File:Radar-velocity firmware rev1.7.3.zip (older: File:Radar-velocity firmware rev1.7.2.zip,File:Radar-velocity firmware rev1.7.1.zip, File:Radar-velocity firmware rev1.6.2.zip, File:Radar-velocity firmware rev1.4.4.zip,File:Radar velocity firmware 1.4.1.zip,File:Radar velocity firmware 1.4.0.zip)

For completeness a BoSL logger script relevant to the provided firmware is provided here: File:BoSL logger rev1.7.1.zip

1. The radar firmware also contains a python script to test that the firmware has been successfully flashed. Open this script to determine if the process was successful. You may need to edit the file to change the COM port which it is using.

After this is done the firmware should be successfully flashed.

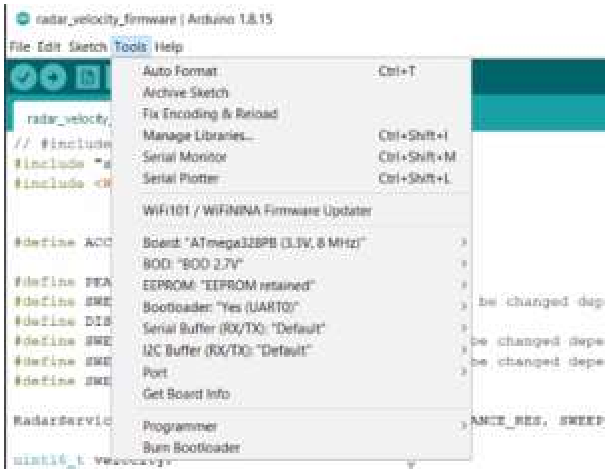

radar_velocity_arduino_settings

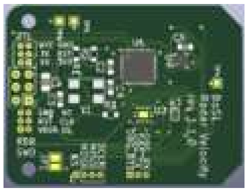

back side of radar PCB

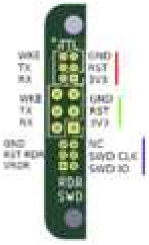

pinout

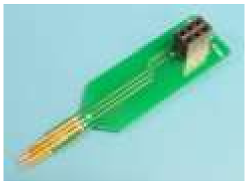

programmer probe

Tips and Tricks

To make a batch of uploading go quicker, the final command in the Arduino IDE terminal can be copied to a command prompt for significantly faster uploading. The file used for uploading however will only be present while the Arduino IDE is open.

3D Printing

Lens

The Lens should be printed out of 100 % infill PLA. Ensure that the 3D printer does not start a new layer on the exterior thread surface. This causes the thread to become warped at places.

Backplate

There are no specific print settings for the back plate.

Body

The body should be printed with a minimum of 60% infill. However layers 1-100 and 301 onwards should be printed at 100% infill.

Testing

Testing should be performed at least at three points during assembly.

- Once after programming,
- Once after the radar pcb has been soldered to the cable
- And once after waterproofing.

Testing after programming and after waterproofing should be done with the testing script provided with the ATmega firmware. Testing after soldering should be done with a multimeter. Each pair of pins on the red connection should be checked to see that they aren't shorted closed, each of the red connection should also have continuity checked with the corresponding wire soldered to the green connection (the end which will be used to connect to the data logger.)

Cabling

Ethernet cable should be used to solder to the sensor, a length of up to 20 m has been confirmed to be working at this time.

1. Strip the cable at one end such that 5mm of copper are exposed on each conductor. The exposed lengths should be as even as possible.
2. Pass the cable through the cable hole on the sensor case body untill at least 100 mm of cable has been pulled through.
3. Solder the cables to the green connection on the radar PCB such that when the PCB is placed in the body, the XM132 will face towards the lens cavity. Solder according to the soldering table, half blue and half brown can be cut off. Ensure that the cable is approximately perpendicular to the PCB
4. Use side cutters to cut off any excess cable.
5. Apply hot glue to the cables around the solder joint, this ensures that the joints are do not bare any stress from cable movements. The hot glue may case plastic strings, ensure that none of these become attached to the XM132 radar IC.
6. Electrically test the connections made as per the testing section.

Pin	Cable
GND	Half Green
RST	Orange
3V3	Green
WKE	Half Orange
TX	Brown
RX	Blue

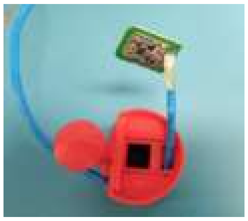

Radar sensor after cable soldering.

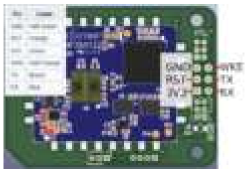

labelled soldering scheme for cable

Waterproofing

Backplate

1. Use a syringe to add some gel-gum near the cavity where the cable from the sensor entered the sensor body. Pull the cable gently back and forth to drive the gel-gum around the cable, creating a seal between the cable and the body.
2. While the gel-gum is still workable, pull the cable back to to fit the PCB into the case, the screw holes on the PCB should align with 2 screw holes in the case body. Use M2 screws and screw down the PCB.
3. If the gel-gum has not filled the cable entry cavity add more until the gel-gum is level with the PCB.
4. Leave the gel-gum to dry.
5. Once dried flip the sensor around to the lens cavity. Carefully use a hot glue to seal the gap between the PCB and the case body. Ensure that the hot glue does not touch the radar IC. Remove any glue strings once dried. This is done to ensure that no gel-gum falls into the lens cavity.
6. Fill the remainder of the back of the radar sensor will gel-gum. Fill with gel-gum until just before it beings to overspill. Wait for any bubbles to be removed from the gel-gum.
7. While the gel-gum is still wet press fit the backplate onto the sensor.

Lens

1. Add some silicone to the internal lens thread of the body
2. Screw the lens down until finger-tight
3. Unscrew the lens and clean off excess silicon on bottom of lens
4. Apply a small amount of silicon to the external thread of the lens
5. Tighten the lens down firmly

6. Use silicon to seal the rim interface between the lens and the body. Only a small amount should be necessary

Video Tutorial

Tutorial 1 for Assembling a Radar Sensor: Introduction

Tutorial 2 for Assembling a Radar Sensor: Remapping ATmega

Tutorial 3 for Assembling a Radar Sensor: XM132 Firmware

Tutorial 4 for Assembling a Radar Sensor: ATmega Firmware

Tutorial 5 for Assembling a Radar Sensor: Sensor Testing

Tutorial 6 for Assembling a Radar Sensor: Soldering Wire

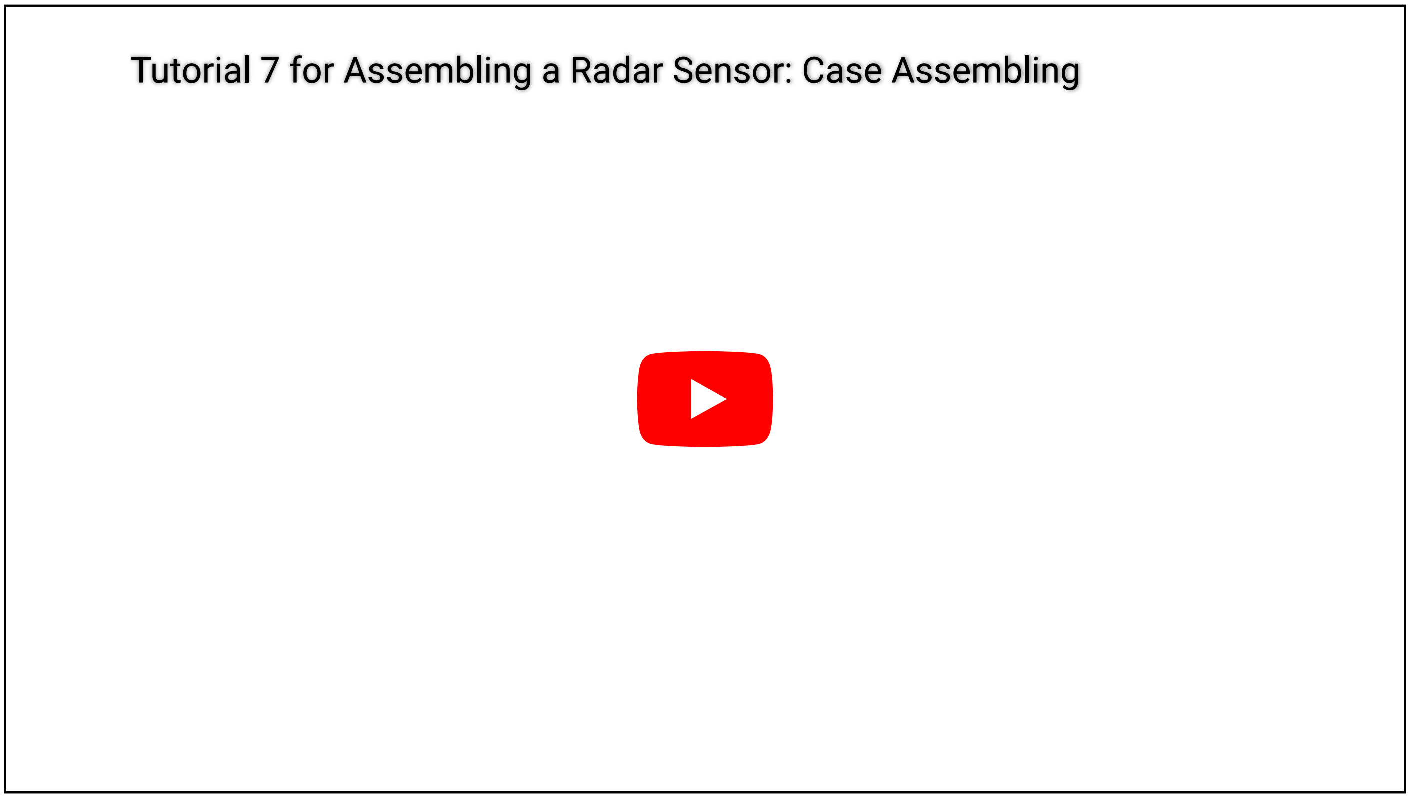

Retrieved from "https://www.bosl.com.au/wiki/index.php?title=Assembling_the_Radar_Sensor&oldid=22626"

This page was last edited on 6 February 2023, at 04:42.

This page has been accessed 276 times.
