# Supplementary material for: A Low-Cost Radar-Based IoT Sensor for Noncontact Measurements of Water Surface Velocity and Depth
Source: Sensors (Basel). 2023 Jul 11;23(14):6314. doi: 10.3390/s23146314 (PMC10385264; doi:10.3390/s23146314)
Supplement: Supplementary file 1 [file radar velocity schematic rev 1.1.1.pdf › supmat/design_files/radar velocity schematic rev 1.1.1.pdf]

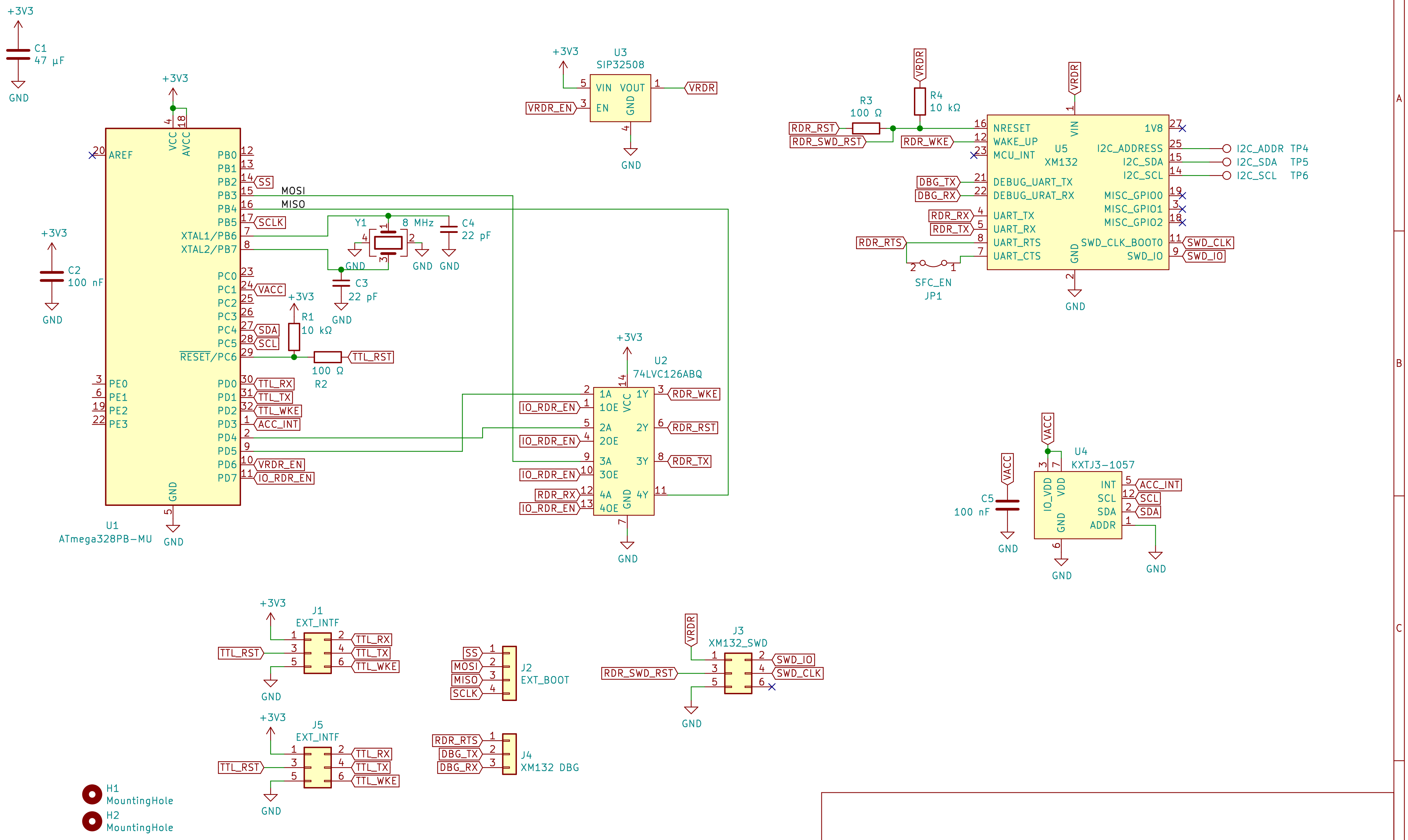

Monash BoSL

Sheet: /

File: EDA.sch

Title: BoSL Radar Velocity

Size: A4 | Date: 2021-09-29 | **Rev: rev 1.1.1**

KiCad E.D.A. kicad (5.1.9)-1 | Id: 1/1
